# Difference Between Posterior Monteggia Fractures and Posterior Fracture‐Dislocation of Proximal Ulna in Adults

**DOI:** 10.1111/os.12784

**Published:** 2020-08-13

**Authors:** Jun‐yang Liu, Ji‐zheng Zhang, Ye‐ming Wang, Xu Tian, Jing‐ming Dong

**Affiliations:** ^1^ Department of Orthopedic Trauma Tianjin Hospital Tianjin China; ^2^ Department of Anesthesiology Tianjin Hospital Tianjin China

**Keywords:** Dislocation of PRUJ, Metaphysis fractures, Posterior fracture‐dislocation of proximal ulna, Posterior Monteggia fractures

## Abstract

**Objective:**

To figure out the difference between patients with posterior Monteggia fractures which were concomitant with proximal radioulnar joint (PRUJ) dislocation and posterior fracture‐dislocation of the proximal ulna that were not concomitant with PRUJ.

**Methods:**

From January 2016 to January 2019, 37 consecutive adult patients who had posterior fracture‐dislocation of proximal ulna (no PRUJ dislocation, n = 16) and posterior Monteggia fractures (PRUJ dislocation, n = 21) were included. All patients had intraoperative fluoroscopy, computed tomography (CT) scans, and standard radiography (anteroposterior view and lateral view). The mechanism of injury, the cases with open fracture, sustained multiple injuries and classification of fracture was recorded. The clinical details of the patients such as the final range of motion (ROM) and the Broberg–Morrey scores were described.

**Results:**

Patients with PRUJ dislocation (ten type A, five type B, and six type D) and those without concomitant PRUJ dislocation (fifteen type A and one type C) exhibited an obvious difference according to the classifications of Jupiter *et al.* (*P* = 0.010). Ninety‐five percent of patients who had PRUJ dislocation were accompanied by a metaphyseal fracture, while only 50% of the patients who did not have PRUJ dislocation were accompanied by a metaphyseal fracture (*P* = 0.002). Meanwhile, 16 of 20 metaphyseal fractures had more than one fragment in the group of dislocations, but five of eight metaphyseal fractures were comminuted in the control group. The two groups exhibited an obvious difference (*P* = 0.009). The 21 patients who sustained a radioulnar dislocation had less mean arc of flexion, pronation, and Broberg–Morrey scores were significantly less than the patients of the control group (flexion: 117.38 ± 14.46 *vs* 127.50 ± 13.416, *P* = 0.035; pronation: 59.76 ± 11.88 *vs* 67.50 ± 6.58, *P* = 0.017; Broberg–Morrey: 80.48 ± 12.17 *vs* 88.19 ± 10.28, *P* = 0.040).

**Conclusions:**

Patients suffering posterior Monteggia fractures had more metaphyseal fractures, more comminuted fractures of the metaphysis, and worse ultimate ulnohumeral motion than patients of posterior fracture‐dislocation of proximal ulna.

## Graphical abstract

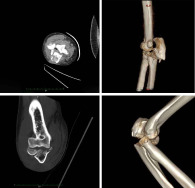

A‐B, posterior Monteggia fractures had comminuted fracture of metaphysis and dislocation of PRUJ. C‐D, posterior fracture‐dislocation of proximal ulna didn't have fracture of metaphysis and no dislocation of PRUJ.

## Introduction

Monteggia fracture is defined as proximal ulnar fracture accompanied by proximal radioulnar joint (PRUJ) disruption^1^. It includes an apex ulna fracture, PRUJ dislocation, and radiocapitellar dislocation. According to Giovanni Battista Monteggia's description in 1814, it referred to a fracture of the proximal third of the ulna accompanied by radial head anterior dislocation from PRUJ and radiocapitellar joints^2^. Monteggia fracture dislocation was classified by Bado into four different types. Type I, II, and III was explained as a dislocation of the radial head dislocation in anterior, posterior, and lateral directions, respectively. Type IV referred to fractures of double bones of the forearm with radial head dislocation^2^. Jupiter and his colleagues subclassified type II posterior Monteggia fractures into four types considering position of ulnar fracture^3^. The ulna fracture has four types: type A, the distal part of the coronoid process and olecranon; type B, metaphysis fracture; type C, diaphysis fracture; and type D, a complicated multi‐fragmented fracture combining some of above levels. Type C of Jupier Classificationis a classical posterior Monteggia fracture which is always accompanied with PRUJ dislocation and radiocapitellar dislocation. But other types of Jupiter Classification are not described whether a PRUJ dislocation exists.

Many authors concluded that the apex posterior fractures of the ulna accompanied by posterior dislocation of radiocapitellar joint, with or without dislocation of PRUJ, are posterior Monteggia fractures or Monteggia‐like lesions^4^, ^5^, ^6^, ^7^. Application of these eponyms to all injuries accompanied by radiocapitellar dislocation and subluxation has brought about many confusions^8^, such as the anterior olecranon fracture‐dislocation with anterior Monteggia fractures, and the posterior olecranon fracture‐dislocation (posterior fracture‐dislocation of the proximal ulna) with posterior Monteggia fractures. Based on the study by Giannicola^9^. these injuries involve six basic lesions which shall be recognized and well‐treated: (i) ulnar fracture; (ii) radio‐humeral dislocation; (iii) ulnohumeral dislocation; (iv) proximal radio‐ulnar dislocation; (v) radial fracture; and (vi) distal radio‐ulnar joint dislocation or interosseus membrane lesion. This theory was accepted and used by other authors^10^, ^11^, ^12^. We think that if there is an apex posterior ulna fracture, dislocation of PRUJ and radiocapitellar dislocation should be called posterior Monteggia fractures, and an apex posterior ulna fracture and radiocapitellar dislocation without dislocation of PRUJ should be called posterior fracture‐dislocation of proximal ulna.

This retrospective study aimed to: (i) evaluate injury patterns, demographics, injury mechanisms of patients and (ii) the short‐run operative treatment results of patients suffering posterior fracture‐dislocation of proximal ulna (with PRUJ dislocation) and posterior Monteggia fractures (without PRUJ dislocation), as well as (iii) confirming prognostic factors impacting the functional result. It is hypothesized in the study that patients with PRUJ dislocation will present a worse final motion and functional score.

## Materials and Methods

### *Inclusion Criteria and Exclusion Criteria*

The patients were eligible for inclusion in this study if they: (i) had proximal ulna fracture, radiocapitellar posterior dislocation with or without proximal radio‐ulnar dislocation; (ii) were surgically treated between January 2016 and January 2019 at our hospital; (iii) equaled to or over the age of 18, and were followed for more than 1 year; and (iv) the related outcomes were completely recorded.

Exclusion criteria were: (i) patients who were followed up for less than 1 year; (ii) patients who did not take pre‐operative radiographs and (iii) patients whose initial operation were conducted in another hospital.

All of the 37 adult patients with 37 elbow fractures and posterior dislocations met the above criteria. All patients had standard radiograph (anteroposterior radiograph and lateral radiograph), computed tomography (CT) scans and intraoperative fluoroscopy, and the mechanism of injury, the patient with open fracture and sustained multiple injuries were recorded.

**Fig 1:**
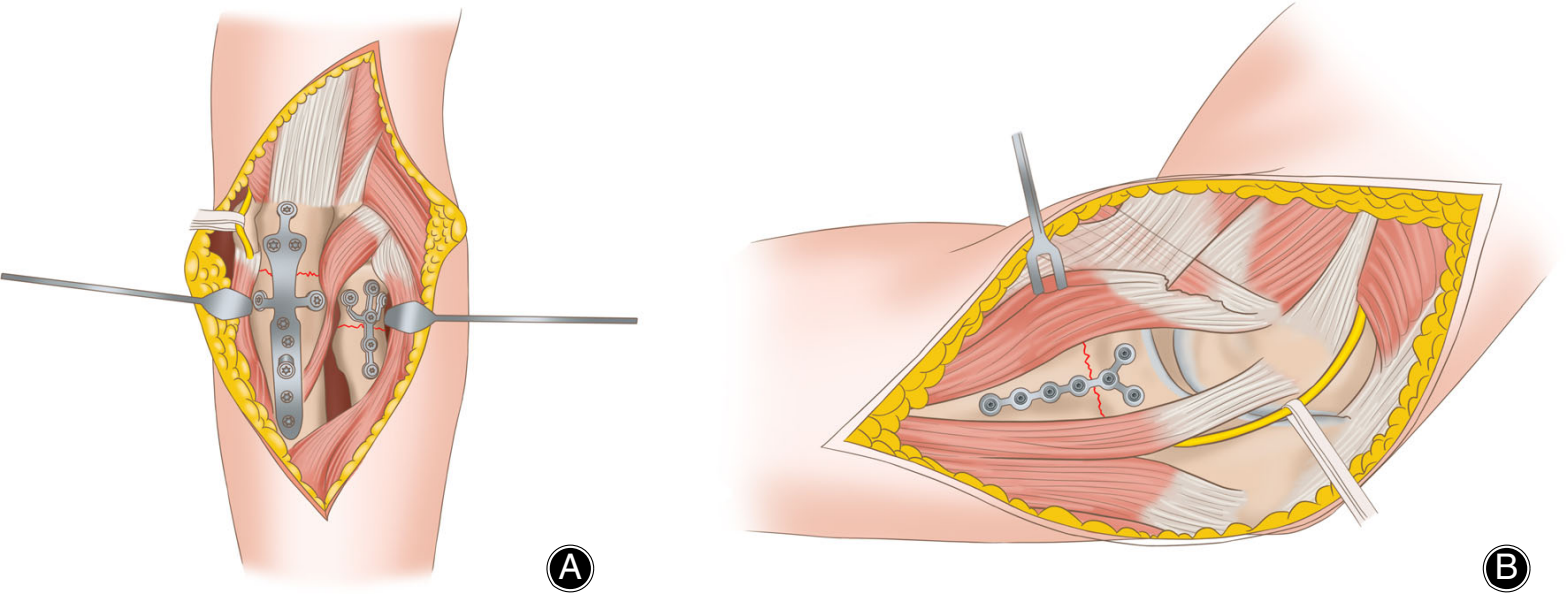
Operation diagram. (A) Posterior approach to fix the ulnar fracture and Kocher approach to fix the radial head fracture. (B) The anteromedial elbow over‐the‐top approach to fix the coronoid fracture.

### *Classification*

Classification of all injuries was performed based on the system proposed by Jupiter *et al* ^3^. Type A fracture was at the olecranon level and entered the ulnohumeral joint. Type B fracture was at the junction of the diaphysis and the metaphysis and in the distal end of the coronoid process. Type C referred to the ulna diaphyseal fracture. Type D involved a fragmented ulna fracture extending from the olecranon to the diaphysis. Some fractures were classified as type D in the paper because the fractures had some fragmentations of coronoid and metaphysis/diaphysis. They could not be classified into any other types.

Mason's classification was used to classify radial head fractures into three types^13^: type 1 is the nondisplaced fracture; type 2 is the displaced partial fracture; and type 3 is the displaced fracture that involves the entire radial head. The classification of Regan and Morrey divided the coronoid process fractures into three grades: Grade I denoted the avulsion of the process tip; grade II denoted a fragment which involved 50% of the process or less; and grade III denoted a fragment that involved over 50% of the process^14^.

The presence of radioulnar joint dislocation, ulnohumeral dislocation, comminution of a coronoid fracture, metaphyseal fracture, comminution of metaphyseal fracture, repairing to lateral collateral ligament, and other informations were also recorded.

### *Treatment*

Patients went into the emergency room. Doctors should check the ipsilateral shoulder, elbow, and wrist. The X‐ray images were taken, which included the full length of the forearm. When treating patients with ulnohumeral joint dislocation and minor coronoid fracture, closed reduction should be performed under general anesthesia, and it would be beneficial to those patients. But, When treating patients with severe fracture injuries, closed reduction should not be performed. Only sling or plaster fixation should be used.

In order to eliminate anesthesia factors, operations under general anesthesia were anesthetized by the same anesthesiologist. Furthermore, patients assigned to collect data were blinded to the anesthesiologist. In the operating room, all patients were monitored with electrocardiogram, pulse oxygen saturation, non‐invasive blood pressure and bispectral index (BIS).

CT examination was completed to evaluate the injury of the bone structure, and the ligament lesions should be assessed after the fractures were fixed in the surgery.

The treatment process of these fractures was performed by five different attending surgeons. All 37 injuries were treated operatively. All patients were placed in the supine position, and posterior approach allowing most of ulnar fractures was used. The Kocher approach was used to fix or reconstruct the radial head. The anteromedial elbow over‐the‐top approach was used if the coronoid could not be fixed from the posterior approach or Kocher approach (when radial head prosthesis was used). There was no transpose or release of the ulnar nerve *in situ* (Fig. 1).

### *Evaluation Methods*

The clinical and radiological data was collected during the follow‐up period.

#### *Range of Motion (ROM)*

Clinically, there were standard procedures to measure the range of motion (ROM) that included extension, flexion, pronation, and supination. The patient should stand upright and anteflect the shoulder to 90°. Extension is measured with the elbow extended as fully as possible with the palm of the hand facing the ceiling in full supination. The patient is then instructed to flex the elbow as fully as possible keeping the humerus parallel to the floor and the forearm supinated. The normal ROM ranges from about 0° to 145°–150°. Pronation and supination are usually measured with the elbows at the side and flexed 90°. Their normal values range around 80° and 90°, respectively.

#### *Broberg–Morrey* *Score and Index*

Broberg–Morrey Score and Index were taken into account for performing the specific evaluation of the study at the last follow‐up. The cohort containing 21 patients accompanied by radioulnar dislocation was regarded as a subgroup. A comparison was performed between the subgroup and the remaining group containing patients without radioulnar dislocation. The rating system of Broberg and Morrey is a 100‐point system, which consists of four sections: motion (40 points), strength (20 points), stability (5 points), and pain (35 points). Scores are as followed: 95–100 points indicates an excellent outcome; 80–94 points, a good outcome; 60–79 points, a fair outcome; ≤60 points, a poor outcome. The outcome can be considered satisfactory if the result is rated as good or excellent, and unsatisfactory if it is fair or poor.

#### *Varus and Valgus Stability*

We tested the varus and valgus stability in the largest extension and 30° of flexion. The posterolateral rotatory stability was evaluated by the pivot shift test^15^, reflected in four grades: normal, mild, moderate, and severely unstable.

#### *Measurement of Radiology*

All patients received the anteroposterior and lateral radiographs on the injured elbow at follow‐up. The radiographs of the degenerative change, capitellar osteopenia, as well as heterotopic ossification were evaluated by a blinded radiologist.

Lamas *et al*.^16^ graded the capitellar osteopenia as severe, moderate, mild, and none. Considering the grading standard of Broberg and Morrey^17^, degenerative changes had four grades: grade 0 meant normal joint; grade 1 was slight narrowing of joint space and minimum formation of osteophyte; grade 2 represented moderate narrowing of joint space and moderate formation of osteophyte; and grade 3 was severe degenerative change with severe joint destruction. The bridging bone showed by the anteroposterior and lateral radiographs defined the union of fractures. Hastings and Graham^18^ defined the heterotopic ossification into seven grades: 0, I, IIA, IIB, IIIA, IIIB, or IIIC.

### *Statistical Analysis*

SPSS22.0 software (IBM Corporation, Chicago, USA) was used for statistical description and analysis of data. Student's *t*‐test was performed on the age, flexion, extension, pronation, supination, and Broberg–Morrey Score. Fisher's exact test was conducted to assess the difference in gender, Jupiter classification, Mason classification, Morrey classification, metaphyseal fracture, and comminution of metaphyseal fracture of the two group. *P* < 0.05 was considered statistically significant.

## Result

### *General Results*

The study involved 25 men and 12 women whose average age was 42.22 years (range, 20–79 years). The average follow‐up time was 27.78 months (range, 12–48 months); 16 patients were injured due to falling from standing heights, 10 patients were injured due to falling from higher heights, eight due to motor vehicle collision, and three due to falling from electric bicycles. One fracture was accompanied by an open wound and grade II according to Gustilo and Anderson^19^.

Patients sustained multiple injuries: there were five patients suffering ipsilateral upper extremity injury (one distal radius fracture, one distal radius and humeral shaft fracture, and three capitellum fractures). There were three patients suffering contralateral upper extremity injury (one severe triad elbow injury‐elbow dislocation accompanied by radial head and coronoid fracture, one forearm fracture, and one distal radius fracture). There were three lower extremity fractures (one femoral neck fracture, one patella fracture, and one tibial plateau fracture), and three sets of rib fractures. There was one abdominal injury. Nerve palsy was not found in any patients.

**Fig 2:**
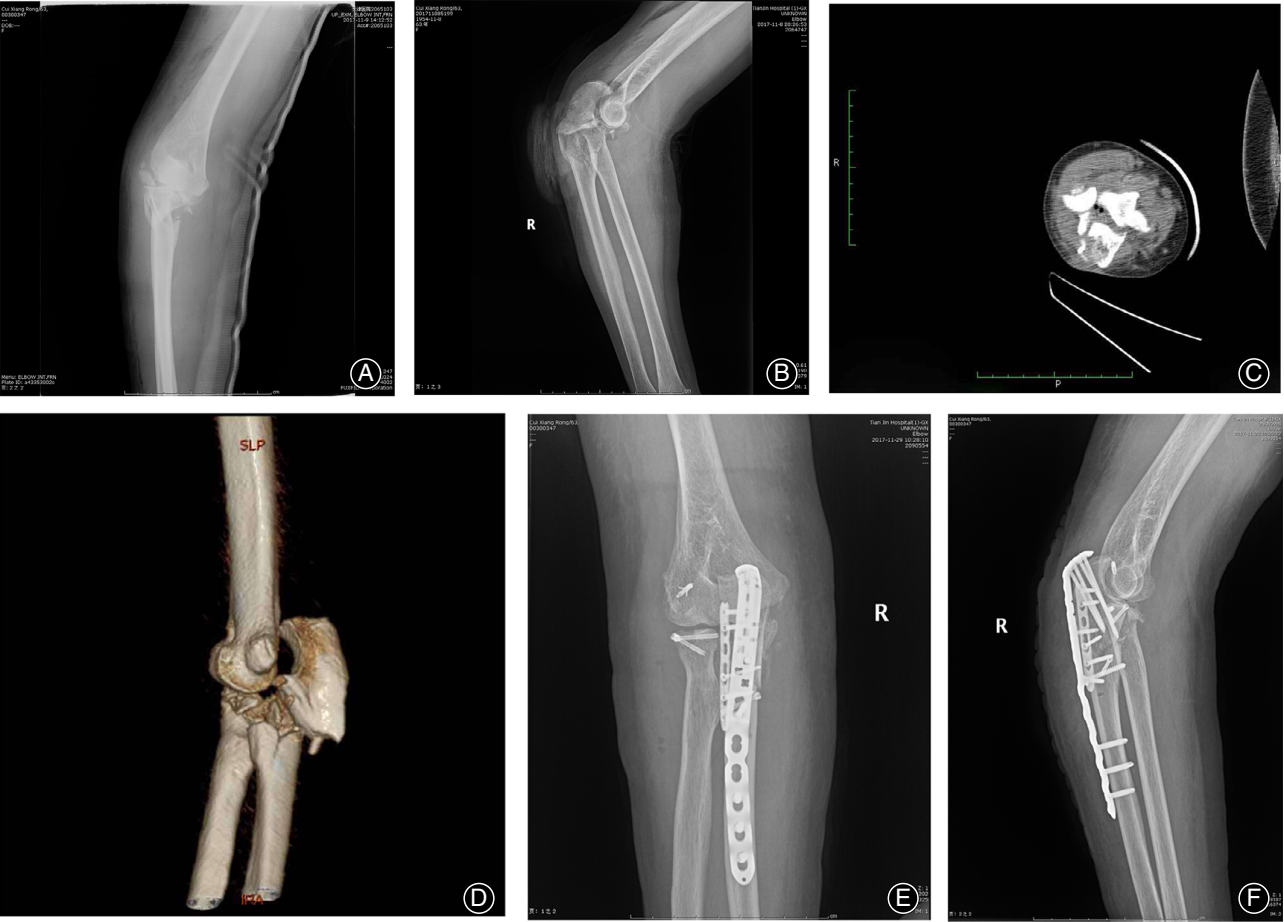
A 63‐year‐old woman falling from a standing height with the right dominant elbow injured. (A, B) Initial anteroposterior and lateral radiograph showed proximal ulna fracture and radial head fracture, accompanied by comminuted metaphyseal fracture and radiocapitellar dislocation. (C, D) A computed tomography image showed the PRUJ dislocation. (E, F) The anteroposterior and lateral radiograph obtained following the operative treatment.

**Fig 3:**
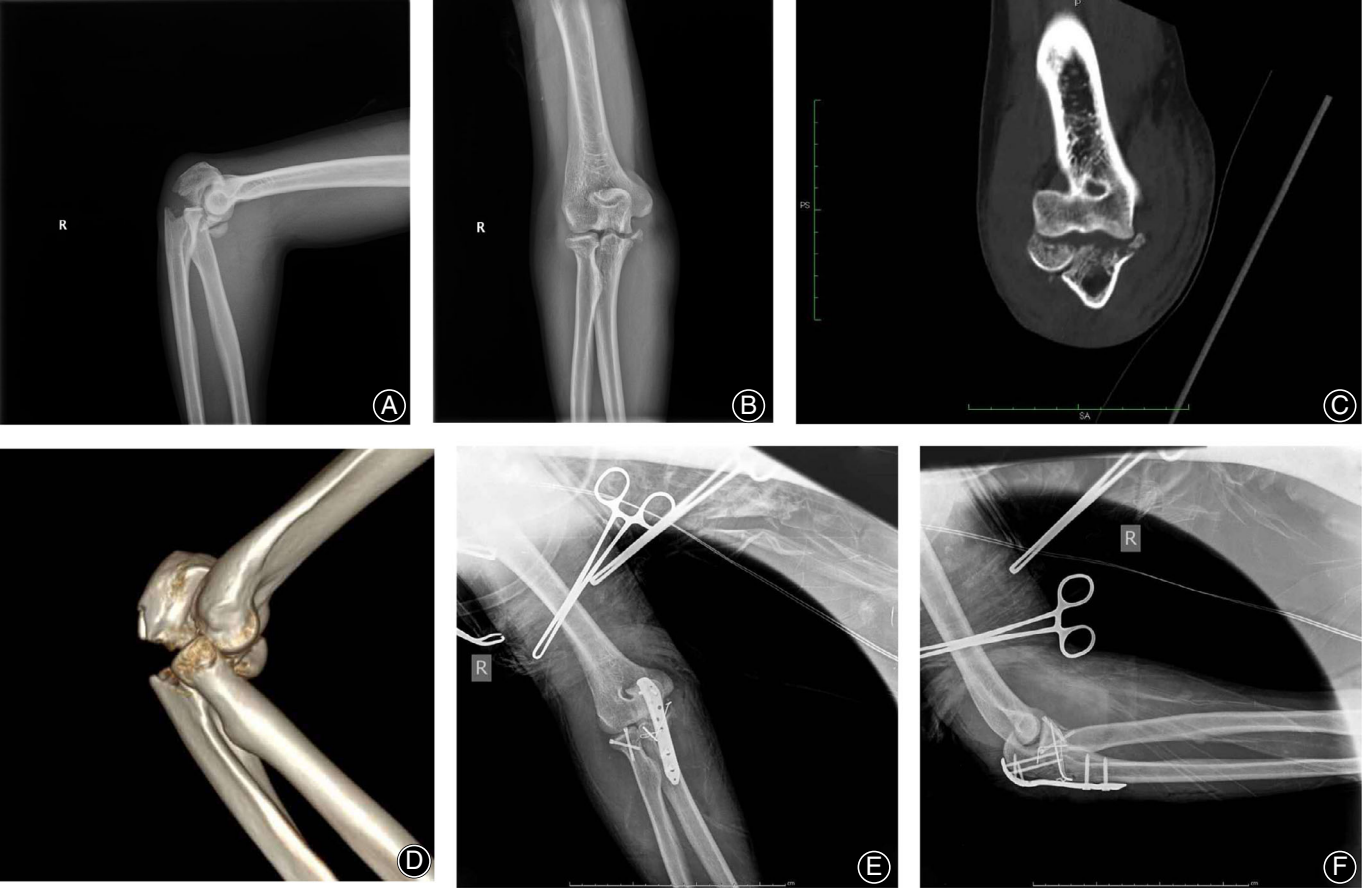
A 30‐year‐old man falling from 1 meter height and injured right dominant elbow. (A, B) Initial anteroposterior and lateral radiograph showed proximal ulna fracture and radial head fracture, with radiocapitellar dislocation. (C, D) A computed tomography image showed that the PRUJ saw no dislocation and no metaphyseal fracture was found. (E, F) The anteroposterior and lateral radiograph that were obtained following the operative treatment.

Twenty‐one patients suffered concomitant proximal radioulnar dislocation (Fig. 2), comprised 13 men and eight women whose average age was 44.71 ± 17.34 years (range, 26–79 years). Sixteen patients did not have concomitant dislocation of PRUJ (Fig. 3), comprised 12 men and four women whose average age was 38.94 ± 11.64 years (range, 20–55 years). The two groups exhibited no obvious difference regarding the average age (*P* = 0.259). Also no difference existed in gender (*P* = 0.399), injury mechanism (*P* = 0.547), and additional injuries (*P* = 0.336).

### *Results of Classifications*

As classified by Jupiter *et al*., there were 25 type A fractures, five type B fractures, and seven type D fractures^3^. Thirty‐six of the 37 injuries (97.3%) suffered radial head concomitant fracture and 22 fractures of type 2 (with partial articular displaced) and 14 fractures of type 3 (with complete articular displaced).

Based on the classification by Jupiter *et al*.^3^, patients who had radioulnar dislocation (10 type A, five type B, and six type D) were different from those patients without radioulnar dislocation (15 type A and one type C) (*P* = 0.010). When the classification was the Jupiter type B and type D, it was more likely to be the posterior Monteggia fracture which was concomitant radioulnar dislocation.

### *Fracture Patterns*

Regarding the coronoid fracture, 33 (89.2%) had coronoid fractures. Twelve patients had fracture of Regan–Morrey type 1, four had fracture of type 2 (<50% of the height of coronoid), and 17 had fracture of type 3 (>50% of the height of coronoid). There were over one fragment in 22 of all coronoid fractures. Fifteen patients had concomitant ulnohumeral dislocation and the two groups exhibited no obvious difference.

All of the patients with dislocation of PRUJ had an associated radial head fracture. The two groups exhibited no obvious difference (*P* = 0.390). But with the radial head fracture in Mason III, the internal fixation and open reduction were worse than the arthroplasty in the function and Broberg–Morrey score. Most patients had coronoid fracture, and both of the groups had no significant difference (*P* = 0.180).

Ninety‐five percent of patients who had PRUJ dislocation were accompanied by metaphyseal fracture, while only 50% of the patients who did not have PRUJ dislocation were accompanied by a metaphyseal fracture (*P* = 0.002). Meanwhile, 16 of 20 metaphyseal fractures were comminuted in the dislocation group, but 5 of 8 metaphyseal fractures were comminuted in the no dislocation group. The two groups exhibited an obvious difference (*P* = 0.009) (Table 1).

**TABLE 1:** Characteristics of the study population

Variable	Dislocation of PRUJ (n = 21)	No dislocation of PRUJ (n = 16)	*P*
Age (year)	44.71 ± 17.34	38.94 ± 11.64	0.259
Sex (male/female)	13/8	12/4	0.399
Jupiter (A/B/C/D)	10/5/0/6	15/0/0/1	0.010
Mason (0/I/II/III/IV)	0/0/14/7	1/0/8/7	0.390
Morrey (0/I/II/III/IV)	5/5/2/9	0/7/2/7	0.180
Metaphyseal fracture	20	8	0.002
Comminution of Metaphyseal fracture	16	5	0.009

PRUJ, proximal radioulnar joint.

Age Values are presented as mean ± SD, *t*‐test. Fisher's exact test helped to compared the variables of sex, Jupiter, Mason, Morrey, metaphyseal fracture and comminution of metaphyseal fracture.

### *Complications*

In the cohort containing 37 patients, seven (18.9%) patients with injured elbows received more than one subsequent treating procedure, four patients were in the group of PRUJ dislocation (two rupture of the plates, one recurrent dislocation, one radial head non‐union), and three patients were in the group of no PRUJ dislocation (one deep infection, one ulnar neuropathy, and one heterotopic ossification) (*P* = 0.357). The patients with complications had worse function and scores than the patients without complication (flexion: 108.57 ± 13.45 *vs* 124.83 ± 13.42, *P* = 0.018; Broberg–Morrey: 68.57 ± 15.79 *vs* 87.37 ± 7.40, *P* = 0.019).

### *Results of Functions*

Among the 37 patients, the average flexion arc and extension were 121.76° (range, 90°–140°), and 16.22° (range, 0°–80°), respectively. The average arc of forearm pronation and average supination were 63.11° (range, 30°–75°) and 66.89° (range, 30°–85°), respectively.

#### *Result of Range of Motion (ROM)*

The 21 patients sustaining a radioulnar dislocation had smaller average flexion arc, pronation, and Broberg‐Morrey score compared with the group suffering no dislocation flexion (117.38 ± 14.46 *vs* 127.50 ± 13.416, *P* = 0.035, pronation 59.76 ± 11.88 *vs* 67.50 ± 6.58, *P* = 0.017). There was similar average extension, supination in the two groups (extension 17.38 ± 10.56 *vs* 14.69 ± 18.03, *P* = 0.632, supination 64.76 ± 16.32 *vs* 69.69 ± 13.23, *P* = 0.323).

#### *Broberg–Morrey* *Score*

Clinical assessment showed significant difference between the two groups (PRUJ dislocation group 80.48 ± 12.17 *vs* no dislocation group 88.19 ± 10.28, *P* = 0.040). The scores of PRUJ dislocation group were significantly less than the scores of no dislocation group.

All patients showed a normal pivot shift and the elbows were stabilized (Table 2).

**TABLE 2:** Clinical outcomes of PRUJ dislocation and no PRUJ dislocation

Variable	Dislocation of PRUJ (n = 21)	No dislocation of PRUJ (n = 16)	*P*
Flexion (°)	117.38 ± 14.46	127.50 ± 13.416	0.035
Extension (°)	17.38 ± 10.56	14.69 ± 18.03	0.632
Pronation (°)	59.76 ± 11.88	67.50 ± 6.58	0.017
Supination (°)	64.76 ± 16.32	69.69 ± 13.23	0.323
Broberg‐Morrey score	80.48 ± 12.17	88.19 ± 10.28	0.040

PRUJ, proximal radioulnar joint.

Values are presented as mean ± SD, *t*‐test.

### *Results of Imageology*

Based on the radiographs, no significant difference existed between the two groups (capitellar osteopenia grade 1, nine patients *vs* grade 1, five patients, *P* = 0.515; degenerative changes grade 1, 17 patients, grade 2, two patients *vs* grade 1, 14 patients, grade 2, zero patient, *P* = 0.643 and heterotopic ossification class I, three patients, class IIA, two patients, class IIIA, zero patient *vs* class I, one patient, class IIA, zero patient, class IIIA, one patient, *P* = 0.887).

## Discussion

Researchers described the classical Monteggia fracture as a proximal ulna fracture accompanied by a radiocapitellar and PRUJ dislocation^1^. Posterior Monteggia fracture was defined as additional traumatic pathologies surrounding the elbow (such as additional radial head fracture, coronoid fracture, or humeroulnar joint dislocation)^20^. But the PRUJ dislocation, as an important characteristic, helps to differ Monteggia fracture from fracture‐dislocation of the proximal ulna^21^.

As is well known, the study was the first one which had patients with clearly defined posterior Monteggia fractures and posterior fracture‐dislocation of the proximal ulna. Based on the present experience review, in these injuries, 21 of 37 fractures (56.8%) were identified with PRUJ dislocation. On the basis of the classification by Jupiter *et al*., the group of posterior Monteggia fractures was 10 of type A, five of type B, and six of type D, but the control group was 15 of type A and one of type D. The fractures with proximal radioulnar dislocation always involved the metaphysis/diaphysis. The proximal radioulnar relationship showed a greater relative sparing when ulna fracture lied in a more proximal position, which was in line with Ring D^22^.

Patients with dislocation of PRUJ had a significantly worse outcome in function (flexion and pronation), and Broberg–Morrey score than patients of the control group. In the dislocation group, two patients had plate fracture and one patient had ulna posterior angulation and recurrent radiocapitellar joint dislocation, because the metaphysis was comminuted and the fractures were unstable. This never happened in the control group.

Posterior Monteggia fractures and posterior fracture‐dislocations of the proximal ulna are complex fractures and dislocations of the elbow joint. There were similarities between them regarding the injury degree of soft issue and bone structure. But PRUJ dislocation, as an essential characteristic, differentiated posterior Monteggia fractures from posterior fracture‐dislocations of the proximal ulna. If ulnar fractures were involved in the metaphysis or diaphysis, the fractures were usually comminuted, and the dislocations of PRUJ more likely to occur. Restoring the anatomical shape of ulna, stable fixation was more difficult, but more important. Finally, when treating complex elbow fractures and dislocations, a definite diagnosis is needed to accurately treat and evaluate the prognosis.

The retrospective nature of the study limited its outcomes. Patients were operated on by different doctors and had relatively short follow‐up time. Besides, results of statistical analysis may present a bias, because of the small number of patients in two subgroups. It limited the ability to draw conclusions. Studies with multiple centers and large sample size should be performed to further confirm the research results.

### *Conclusion*

In conclusion, there are similarities between posterior Monteggia fractures and posterior fracture‐dislocations of the proximal ulna in fracture of proximal ulnar and dislocation of radiocapitellar joint. But posterior Monteggia fractures have dislocation of PRUJ and have more comminuted fracture of metaphysis than patients of posterior fracture‐dislocation of proximal ulna. Accurate diagnoses of these complex elbow fracture‐dislocation help doctors to provide effective treatments and evaluate prognosis.
